# Biosecurity measures to reduce influenza infections in military barracks in Ghana

**DOI:** 10.1186/s13104-014-0956-0

**Published:** 2015-01-23

**Authors:** Prince Godfred Agbenohevi, John Kofi Odoom, Samuel Bel-Nono, Edward Owusu Nyarko, Mahama Alhassan, David Rodgers, Fenteng Danso, Richard D Suu-Ire, Joseph Humphrey Kofi Bonney, James Aboagye, Karl C Kronmann, Chris Duplessis, Buhari Anthony Oyofo, William Kwabena Ampofo

**Affiliations:** Ghana Armed Forces Medical Directorate, Accra, Ghana; Department of Virology, Noguchi Memorial Institute for Medical Research, Legon, Accra, Ghana; Veterinary Services Directorate, Accra, Ghana; Wildlife Division, Forestry Commission, Accra, Ghana; U.S. Naval Medical Research Unit No.3, Cairo, Egypt

**Keywords:** Backyard poultry, Pandemic avian influenza, Biosecurity, Education, Military, Ghana

## Abstract

**Background:**

Military barracks in Ghana have backyard poultry populations but the methods used here involve low biosecurity measures and high risk zoonosis such as avian influenza A viruses or Newcastle disease. We assessed biosecurity measures intended to minimize the risk of influenza virus infection among troops and poultry keepers in military barracks.

**Findings:**

We educated troops and used a questionnaire to collect information on animal populations and handling practices from 168 individuals within 203 households in military barracks. Cloacal and tracheal samples were taken from 892 healthy domestic and domesticated wild birds, 91 sick birds and 6 water samples for analysis using molecular techniques for the detection of influenza A virus. Of the 1090 participants educated and 168 that responded to a questionnaire, 818 (75%) and 129 (76.8%) respectively have heard of pandemic avian influenza and the risks associated with its infection. Even though no evidence of the presence of avian influenza infection was found in the 985 birds sampled, only 19.5% of responders indicated they disinfect their coops regularly and 28% wash their hands after handling their birds. Vaccination of birds and use of personal protective clothing while handling the birds were low putting the people at risk.

**Conclusion:**

Though some efforts have been made to improve biosecurity practices, interventions that help to protect the poultry flock from direct contact have to be practiced. Basic hygiene like washing of hands with soap and running water and regular cleaning of chicken coops are needed to prevent the spread of diseases among birds and between birds and humans.

## Findings

### Introduction

Since the emergence of the highly pathogenic avian influenza (HPAI) virus subtype H5N1 in 1997 in Hong Kong [1,2] and its subsequent re-emergence in ensuing years, 648 laboratory confirmed human cases with influenza A(H5N1) have been reported with at least 384 deaths from 2003 through 20 December 2013 [3]. Another subtype of avian influenza that has made the headlines is A(H7N9). As of November 2013, 142 confirmed cases of human infection with avian influenza A(H7N9) have been reported to the World Health Organisation (WHO) by the China National Health and Family Planning Commission [4]. Avian influenza (AI) virus accounts for the death and culling of millions of domestic poultry globally, impacting negatively on economic growth and food security. Poultry represents an important sector in animal production, with backyard flocks representing a huge majority, especially in the developing countries. In these countries, individuals raise poultry to meet household food demands and as additional source of supplementary income for livelihood [5,6]. Backyard poultry is characterized by small flocks with low biosecurity measures often consisting of free indigenous unselected breeds of various ages, with various species mixed in the same flock [7-10]. Backyard production methods using traditional husbandry practices, poor housing, overcrowding and close proximity to human habitation lead to high risk of infectious diseases, including zoonosis such as Newcastle Disease and HPAI [11].

*Anseriformes* (ducks, geese, swans) are a natural reservoir for influenza and play a major role in influenza transmission (11). Though current studies in West Africa [12] indicate that there is no AI in backyard poultry, Egypt is still facing recurrent HPAI (H5N1) outbreaks [13]. Studies which identify exposure as an important setting also report backyard settings as a major contributor of cases [14]. Among the risk factors identified for H5N1 human infections were close or direct contact with poultry and transmission via contaminated environment. Notable among these risk factors were direct contact with infected blood or body fluids during slaughtering, removal of feathers and organs, washing of meat, feeding and caring [15]. Related factors connected to environmental exposure to HPAI include: cleaning infected poultry houses, removal of faeces from infected birds, using poultry waste as fertilizer, inhalation of contaminated dried faeces and ingestion and/or intranasal inoculation of contaminated water. This increasing risk has led to a review of pandemic preparedness plans and their potential shortcomings for Africa [16] and Ghana [17]. Shortly after the pandemic a preparedness plan in Ghana was put together, three outbreaks of AI were recorded among birds in poultry farms [18,19] close to military barracks with no human case.

We recently educated troops and sampled their birds for avian influenza infection. As part of poultry raising activities, raisers had close contact with their poultry including touching them while putting them into sheds, feeding sick poultry by hand, killing, defeathering and butchering poultry. Though no avian influenza circulation was found and participants demonstrated good knowledge of pandemic avian influenza, biosecurity practices were poor. In the present study we determined to strengthen education on biosecurity practices and associated risk to reduce influenza infection in military camps in the country.

## Methods

### Study design and setting

**Figure 1:**
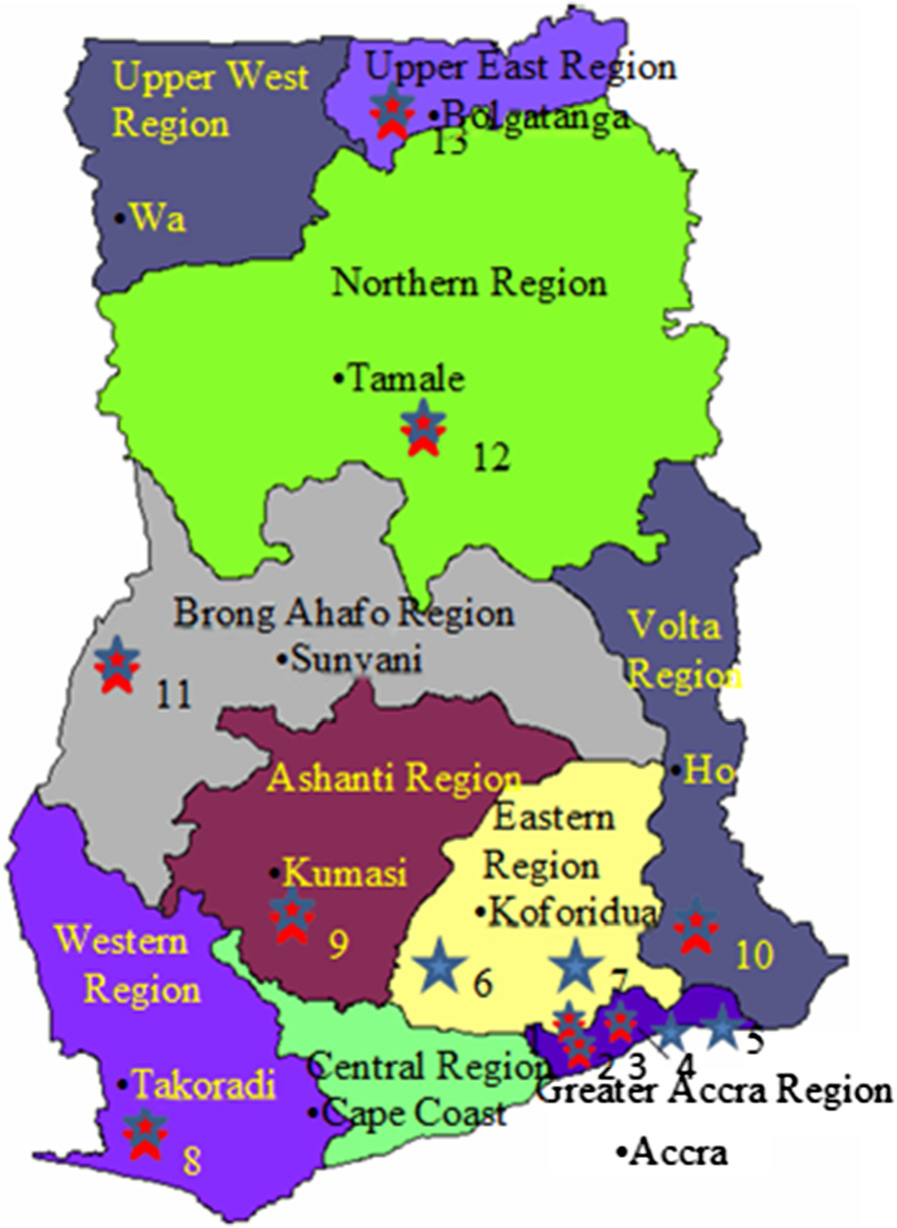
**Regional map of Ghana showing the distribution of military barracks visited.** “Red star symbol” Military barracks where both sampling and education was done; 1; 5BN, 2; 49 Engineer Regiment, 3; 1BN, 8; 2BN, 9; 4BN, 10; 66 Artillery, 11; 3MRS, 12; Airforce/Airborne, 13; Bazua. “Blue star symbol” Military barracks where only sampling was done; 4; Shai Hills, 5; Naval Base, 6: Asutuare, 7; Achiase.

Seminars and sampling took place in 13 GAF barracks in the country from 5 to 29 March 2012. These barracks which cover the country’s vegetation zones of coastal, tropical rain forest and northern savannah belts are located across the length and breadth of the country (Figure 1).

### Education

Educational seminars on AI were held to further strengthen the existing knowledge and increase the biosecurity measures around the military barracks of the GAF. The education highlighted biosecurity measures including cleaning and disinfection combined with vaccination and strategic treatment and bird management when sick or dead. The beneficiaries included the Army, Navy, Air Force and their dependants as well as civilian employees of the Ministry of Defence living within or near the barracks. The program was launched at the Burma Hall of Burma Camp on March 5, 2012 for all ranks of GAF in general and the Medical Department in particular. Families and dependants of troops, teachers and school children from GAF schools were in attendance. Resource persons included scientists from the Noguchi Memorial Institute for Medical Research (NMIMR), officers of the Veterinary Services Directorate (VSD) and personnel from the Wildlife and National Disaster Management Organisation (NADMO). Subsequent seminars in other garrisons covered officers and their families, school children and teachers. Details of attendance are as shown Table 1. During the seminars, emphases was laid on poultry raising to desist from close contact with their poultry including touching them while feeding and putting them into pens, and feeding on sick poultry. Raisers were advised to seek help from veterinary officers and apply the services of butchers for killing their poultry.

**Table 1:** **Attendance of participants during troop and student education in military barracks visited**

	**Garrison**	**Barracks**	**Attendance**			
**Region**			**Troops**		**Students**	**Total**
			**Male**	**Female**		
Greater	1 & 5	Teshie	37	19	0	56
		Burma Camp	158	47	0	205
		Michel Camp	69	11	0	80
Volta	7	66 artillery	28	21	0	49
Western	2	Myohaung Barracks	65	8	83	156
Ashanti	4	Wadara	119	12	0	131
Brong Ahafo	3	Liberation Barracks	106	37	108	251
Northern	6	Airforce/borne	49	2	87	138
Upper East		Bawku/Bazua	24	0	0	24
Total			655	157	278	1090

### Sampling

Using an active avian influenza surveillance approach, a descriptive cross sectional study was conducted within 13 military barracks. A simple random sampling procedure was used to select households in the barracks. Households were classified according to the installed capacity in the country [19]. Using criteria for eligibility, birds were conveniently selected for either tracheal or cloacal swabbing based on whether subjects were apparently healthy, had respiratory signs or gastroenteritis or with nervous illness. Verbal consent was obtained from all backyard poultry farm owners or care takers to take swabs from their birds. Backyard poultry owners and household members were interviewed to explore their understanding of poultry illnesses, caring for birds and biosecurity practices. A semi-structured questionnaire was administered and information on demographics, basic hygienic practices and quantity of poultry owned were sought. In addition, respondents were asked about health seeking behaviour for their animals including use of available veterinary services to determine causes of death and reporting of sick birds on their farms, knowledge of the cause of death, and knowledge of avian influenza.

A total of 985 birds made up of 892 healthy, 91 sick and 2 dead birds from 203 households were sampled and 168 questionnaires administered (Tables 2 and 3). In addition, 6 water samples common to domesticated and wild birds were sampled. All samples collected were appropriately labelled, stored and transported in cold boxes containing frozen ice packs to the National Influenza Centre (NIC), NMIMR for processing. At the laboratory, all samples were transferred to −70°C for storage until ready for processing.

**Table 2:** **Regional bird census in military barracks**

**Region**	**Barracks**	**Bird population**	**No of households**	**No of birds sampled**			**Questionaires administered**	**Feed or water sampled**
				**Healthy**	**Sick**	**Dead**		
**Greater Accra**	Burma Camp	267	24	126	19	2	21	0
	Teshie	377	22	89	14	0	17	0
	Michel Camp	461	21	92	6	0	12	0
	Naval Base	284	11	42	2	0	6	3
	Shai Hills	239	6	35	6	0	3	0
**Eastern**	Asutuare	100	1	15	0	0	0	0
	Achiase	90	4	17	0	0	3	3
**Volta**	Ho	126	6	19	8	0	7	0
**Western**	Takoradi	617	28	93	2	0	30	0
**Ashanti**	Kumasi	238	12	77	6	0	11	0
**Brong Ahafo**	Sunyani	834	23	132	21	0	24	0
**Northern**	Tamale	1509	28	105	6	0	27	0
**Upper East**	Bawku/Bazua	112	17	50	1	0	7	0
**Total**		**5254**	**203**	**892**	**91**	**2**	**168**	**6**

**Table 3:** **Different species of healthy poultry sampled within military barracks**

**Region**	**Barracks**	**Bird species**							**Total**
		**Duck**	**Fowl**	**Goose**	**Guinea fowl**	**Mallard**	**Pigeon**	**Turkey**	
**Greater Accra**	Burma Camp	26	87	2	0	3	0	8	**126**
	Teshie	8	75	2	0	0	0	4	**89**
	Michel Camp	27	59	0	1	0	0	5	**92**
	Naval Base	8	34	0	0	0	0	0	**42**
	Shai Hills	5	30	0	0	0	0	0	**35**
	Asutuare	0	15	0	0	0	0	0	**15**
**Eastern**	Achiase	8	9	0	0	0	0	0	**17**
**Volta**	Ho	0	14	0	0	0	0	5	**19**
**Western**	Takoradi	5	87	0	0	0	0	1	**93**
**Ashanti**	Kumasi	8	52	0	0	0	0	17	**77**
**Brong Ahafo**	Sunyani	15	82	0	12	0	0	23	**132**
**Northern**	Tamale	1	72	0	22	0	3	7	**105**
**Upper East**	Bawku/Bazua	3	31	0	13	0	3	0	**50**
**Total**		**114**	**647**	**4**	**48**	**3**	**6**	**70**	**892**

### Sample treatment

Processing of samples took place in the Biosafety level-3 laboratory. Samples were pooled according to sample type (tracheal or cloacal), bird type; healthy, sick, dead and household. In all, a total of 125 pools from healthy (105), sick (17) dead (1) and water (2) were obtained. The pools from 91 fowls found with pox-like lesions and respiratory abnormalities and 2 dead birds were first separated and processed followed by the pools from healthy birds. The pools ranged from a single sample to maximum of 7.

### RNA Extraction and rRT-PCR

RNA extraction and real-time PCR were as described before [19]. Briefly, viral RNA was extracted from 140 μl of bird and water samples using the QIAmp viral RNA mini kit (Qiagen, Hilden, Germany) according to the manufacturer’s instructions. RNA was eluted in 60 μl of elution buffer and 8 μl used as template for real time Reverse Transcription-Polymerase Chain Reaction (rRT-PCR). Two rRT-PCR protocols described by the Centers for Disease Control and Prevention (CDC), Atlanta, Georgia, USA and Spackman *et al*. [20], for influenza viruses, were used to screen all the samples [16,17]. RNA was extracted from swab, cloaca and water samples and amplified by rRT-PCR using the AgPath-ID One-Step RT-PCR Kit (Ambion, Austin, Texas, USA) in a 25 μl reaction mixture or Qiagen One Step RT-PCR Kit (Hilden, Germany). Using specific primer and probe sets for Newcastle Disease, RNA from sick birds were also screened.

### Statistical analysis

Demographic data was entered in an electronic database file (Microsoft Excel, 2003). Basic analyses were performed using Microsoft Excel to generate frequencies, graphs and tables. Data were analysed using three statistical tests (i) student t-test, (ii) Pearson’s chi square and (iii) Statistical Package for the Social Sciences (SPSS) 17.0 to generate percentages and p-values.

### Ethical approval

Ethical approval for the surveillance of influenza virus in acute respiratory illness in Ghana was obtained from the Institutional Review Board (IRB) of Noguchi Memorial Institute for Medical Research.

## Results

A total of 1090 persons comprising of troops, their spouses, dependents and students were educated. As shown in Table 1, 655 (61%) male troops and 278 (25.5%) students attended the programme. Troops from the 13 barracks visited participated in the seminars as against students from only 8 schools within 3 barracks. The highest attendance of 205 (18.8%) troops was recorded in Burma Camp while the Liberation Barracks recorded the highest number of 108 students. Even though Burma Camp recorded the highest female troop participation, total female attendance was low (14.4%). Interaction with the troops revealed that 668 (65%) have heard of pandemic avian influenza and the risks associated with its infection. They have objectively good knowledge of pandemic avian influenza, symptoms of the disease and the effects of infection of the virus. Intelligent questions were asked by the students for clarification or better understanding of the biosecurity measures.

Of the 203 households visited, 892 samples made up of 778 tracheal and 114 cloacal samples were taken from healthy birds. In addition, 2 (0.2%) dead birds were sampled from Burma Camp while 6 water samples, three each from Achiase and Naval Base were taken from sources of drinking water for domestic birds that was also exposed to wild birds. Tables 2 and 3 depict the different bird species sampled and the bird population census carried out in the military barracks. Sunyani recorded the highest bird population as compared to Achiase with the lowest. Healthy fowls (*Gallus gallus domesticus*) 647 (72.5%) and ducks (*Anas platyrhyncos domesticus*) 114 (13%) were commonly found in every garrison. Healthy turkeys (*Meleagris gallopavo*) 70 (8%) were found in 8 of the 13 garrisons visited. Some wild birds like the mallard duck (*Anas platyrhyncos domesticus*) were also sampled at Burma Camp. Furthermore, tracheal and cloacal samples from 91 (9.2%) sick birds from 11 garrisons with fowl pox lesions and upper respiratory tract infection were collected with the bulk from Sunyani and Burma Camp. Free ranging of poultry, mixing of poultry from different households and with wild birds and their close interaction with humans was a common phenomenon.

Besides, none of the samples from the sick, dead or healthy fowls were positive for influenza A using the two real-time RT-PCR protocols. Similarly, the water samples also tested negative for influenza A. However, 2 sick fowls from Myohaung Barracks tested positive for Newcastle disease.

A total of 168 questionnaires were administered to 203 households keeping backyard poultry. More females 142 (70%) than male respondents completed the questionnaire. Of these, 129 (76.8%) have heard of pandemic avian influenza and the risks associated with its infection. All the respondents either feed the poultry or sweep the poultry droppings. Most (64%) of them had heard about avian influenza infection (p = 0.05) either through the television (32.1%), radio (31.5%), newspapers (31.5%) or by other means (4.9%) which include durbars, friends and posters (Table 4). Of those who had heard of avian influenza, 126 (p < 0.001) had good knowledge of the different types of influenza viruses. Nevertheless, only 52 (25.6%) said avian influenza could be acquired in all species of bird.

**Table 4:** **Source of participants information on avian influenza and knowledge about protective behaviour among poultry keepers in military barracks**

	**N = 168**	
	**No (%)**	**P-value**
Where did you hear of AI		0.05
TV	54 (32.1)	
Radio	53 (31.5)	
News paper	53 (31.5)	
Durbars	5 (3.0)	
Poster	3 (1.9)	
How often do you use gloves		<0.001
Never	160 (95)	
Always	3 (2)	
Occasional	5 (3)	
Do you use boots		<0.001
Never	162 (96)	
Always	2 (1)	
Occasional	4 (2)	
How often do you use face mask		<0.001
Never	164 (98)	
Always	3 (2)	
Occasional	1 (1)	
How often do you wash your hands during bird handling		<0.001
Never	112 (68)	
Always	46 (28)	
Occasional	10 (6)	
How often do you disinfect your coop		0.066
Regular	32 (19.5)	
Seldom	84 (50.0)	
Never	52 (30.5)	

Varied knowledge was expressed on the mode of transmission: majority (69%) said it could be from bird to bird while others (15%) attributed it to bird to man through improper handling of infected bird. Most (60%) people kept poultry with poor husbandry practices such as no disinfection of the coops. Despite the fact that respondents were aware of cleaning and disinfection of their hen coops, only 19.5% disinfect their hands regularly while 50% disinfect occasionally and 30.5% never disinfect but the difference in disinfection among respondents did not reach statistical significance (Table 4). Knowledge on disposal of dead birds also varied from throwing away (42.3%), burying (19%), removing (32%) to burning (1%). Only 3% of respondents claim to eat birds when sick or dead. A few (5%) of respondents answered ‘yes’ for health seeking behaviour for their poultry including use of available veterinary services but vaccination of birds was not a practice.

On safety issues, only 28% reported to always wash their hands after bird handling. As shown in Table 4, there was statistical difference (p < 0.001) in how respondents apply gloves (5%), face mask (3%) and boots (3%) when handling their birds.

## Discussion

Poultry keeping in military barracks dates back to the introduction of “Operation Feed Yourself” programme by the Military government of General Kutu Acheampong after the 1972 Coup d’état. The programme introduced subsistence farming activities involving food and animal production in barracks. The surge in these activities, especially poultry farming, brought up the likely potential of exposure of the inhabitants of barracks to the risk of avian influenza infection considering the close proximity of backyard birds to homes. Proper biosecurity measures became necessary in lieu of these activities and with the three recorded outbreaks of AI near military barracks.

In this study, military personnel and their dependents from 13 barracks and children from 8 schools were educated on biosecurity measures associated with backyard poultry. Of the 1090 participants that attended the seminar, only 3 had been educated previously [19]. This was as a result of their frequent rotation and assignment on missions which necessitates continuous education of the troops. It was observed during the seminars that less female troops and spouses attended the program, nevertheless, those present contributed to the discussions. School children from eight schools only within three barracks participated in the seminars, which could be attributed to inadequate dissemination of information to the schools to release the children to attend the program. In most homes, children and women were those who take care of the birds including provision of water, food, cleaning, maintenance of the coops, de-feathering and slaughtering of the birds and processing of fresh meat. These roles expose them to higher risk and moreover they hardly wash their hands after such works.

During the discussions, the school children wanted to know the mode of transmission and how they could protect themselves against infection. This made their inclusion very important as such seminars educate them on the disease and its preventive measures. The results as presented here and elsewhere [21] indicate that education provides knowledge and motivation to people at high risk of H5N1 infection and enables them to take measures to reduce the risk. Analyses from the questionnaire showed that respondents gained knowledge on AI through TV, radio and newspapers with no statistical difference. These observations have been reported by other studies [22-24]. The findings further suggest a beneficial effect of the mass media in information dissemination. However, we also observed during the seminars that even though troops were adequately informed about avian influenza, symptoms of the disease, its spread and prevention, majority (p < 0.001) of them do not put the knowledge into practice. This was obvious when biosecurity measures like wearing of gloves, boots and respirators were found to be virtually non-existing. Poultry keepers were not keen on the use of personal protective equipment but rather perceive it as additional unaffordable cost due to the economic constraints. A similar observation was made in Nigeria and Nepal [22,23] which is contrary to the practice in Italy where considerable higher rates of protective clothing by poultry raisers were recorded [25]. Our data also showed that washing of hands before and after poultry handling was low (28%). This basic hygienic practice can be strengthened through continuous education. There was substantial difference on how poultry coops are disinfected. As some respondents use water, sand, saw dust, ash, disinfectant, soap and bleach, most (30.5%) never disinfect their coops. Reduced rate of disinfection therefore exposes the poultry raisers and their families to increased risks. Sweeping of the coops was however habitual with high frequency as found elsewhere [25].

A total of 91 (9.2%) sick birds were found in all barracks except Achiase and Asutuare during the household visit. Most of these poultry were kept together with the healthy ones. This poses another risk as one sick bird can infect other healthy birds. No medical attention is sought for the sick birds but is left at the mercy of the prevailing conditions to either recover or die. A few of the respondents reported slaughtering the birds for meal when the bird was about to die while others reported selling the sick birds. A few however reported burying the birds when they die. These results indicate that, few participants maintained traditional habits of eating sick poultry and did not have sufficient knowledge about the risks of H5N1 infection, with economic difficulties possibly being a contributing factor for these behaviours. This finding provides evidence that awareness does not necessarily lead to behaviour change. Behaviour change includes broad and multidisciplinary intervention, which combines communication, realistic and useful recommendations, including economic considerations. While knowledge that disease can spread from sick birds to humans is common, education is needed on how to minimize risk of disease spread amongst bird populations, and from birds to humans, as well as understanding what to do with sick birds. This findings, and other reports [26,27], further provide evidence that continuous education and training is a process of updating knowledge, developing skills, bringing about attitudinal changes, and improving the knowledge and skills of troops who may be called upon in a pandemic to perform their tasks efficiently and effectively.

We sampled 983 birds, from 7 different species and tested for influenza A virus. Whereas fowls were common and mostly found in every household, guinea fowls were confined to the northern sector of the country. Apart from 91 fowls found with respiratory abnormalities, all the birds were healthy. Cloacal and tracheal samples from these birds and water samples subjected to influenza A virus testing using two real-time PCR protocol were all negative for influenza A. This findings show no evidence of the presence of AIV in the birds sampled. The results confirm our earlier findings of no AI virus circulation in the military barracks and further agree with a recent study carried out in West Africa [12] where no influenza virus was detected from swabs and blood samples collected during active influenza virus surveillance. With the free ranging of birds common in all the barracks, available data have shown free ranging practices to enable easier and cheap access to feed on the ground or water from ponds or rivers [28].

Some of the low biosecurity practices including close proximity of chicken coops to residents’ windows and no fencing of chicken coops that was earlier on observed by the group have been dealt with. Due to these efforts in biosecurity practices observed, the poultry keepers were supplied with respirators, gloves and farm coats and the GAF was advised to also provide boots, hand wash basins at strategic sites and possibly foot bath for poultry keepers to adequately protect them from the risk of infection. Continuous education in the form of workshops by the GAF is essential to update troops and poultry keepers on basic hygiene, proper quarantine and better reporting of sick birds to the appropriate authorities. As the GAF is an essential component of the biosecurity and pandemic response for Ghana, their vulnerability to outbreaks of diseases can endanger their capability to provide stability in times of crisis.

## Conclusion

This study shows some improvement in biosecurity practices, moderate attitudes and practices with good knowledge related to avian influenza among troops and poultry keepers. Our findings could provide scientific support to assist the Ghana Armed Forces in developing strategies and health education campaigns to prevent transmission of the AI virus among the backyard poultry raisers and the general public. In the face of emerging influenza viruses, avoidance of direct contact with sick, dying or dead poultry, the use of protective equipment such as gloves and face masks when contact is unavoidable and the application of basic hygiene such as good cleaning of chicken coops and washing of hands with soap and running water after poultry handling is highly recommended to reduce the spread of AI viruses. Furthermore, bio-security policy formulation should be initiated for poultry raisers to safeguard life and enhance performance and quality of poultry production.
